# Blood Pressure and Pleth Variability Index as Predictors of Tourniquet-Release Hypotension in Elderly Patients Undergoing Total Knee Arthroplasty: A Prospective Observational Study

**DOI:** 10.3390/life16060973

**Published:** 2026-06-09

**Authors:** Sangho Lee, Jung Eun Kim, Yeji Yang, Harin Hong, Hee Yong Kang

**Affiliations:** Department of Anesthesiology and Pain Medicine, Kyung Hee University College of Medicine, Kyung Hee University Hospital, Seoul 02447, Republic of Korea; silzzang15@naver.com (S.L.); geri200@khu.ac.kr (J.E.K.);

**Keywords:** blood pressure, hypotension, pleth variability index, elderly patients, total knee arthroplasty, tourniquet

## Abstract

**Background**: Tourniquet release during total knee arthroplasty (TKA) can cause abrupt hypotension in elderly patients, but simple intraoperative predictors remain unclear. We evaluated whether blood pressure and the pleth variability index (PVi) predict tourniquet-release hypotension. **Methods**: In this prospective observational study, 90 elderly patients undergoing TKA with a thigh tourniquet were analyzed. Noninvasive blood pressure and PVi were recorded at predefined perioperative time points. The primary endpoint was hypotension after deflation, defined as mean blood pressure < 65 mmHg. Secondary exploratory endpoints were systolic blood pressure < 90 mmHg and a ≥20% decrease in systolic blood pressure from pre-release values. **Results**: The primary endpoint occurred in 28.9% of patients and was more common in those with lower pre-release blood pressure. In multivariable analysis, pre-release mean blood pressure and PVi measured immediately after intubation independently predicted hypotension, with odds ratios of 0.95 per 1 mmHg increase and 1.12 per 1-point increase, respectively. The combined model showed moderate discrimination (AUC = 0.71). Similar patterns were observed for systolic definitions, without clear associations with early postoperative complications or hospital length of stay. **Conclusions**: Lower pre-release mean blood pressure and higher intubation PVi may help identify elderly TKA patients at risk of tourniquet-release hypotension.

## 1. Introduction

Total knee arthroplasty (TKA) is frequently performed in elderly patients, many of whom have long-standing hypertension, coronary artery disease, and other cardiovascular comorbidities [1]. Pneumatic thigh tourniquets are commonly used to reduce blood loss and improve surgical visualization, but tourniquet deflation may cause abrupt hemodynamic changes, including hypotension, acidosis, and hyperkalemia [2]. In vulnerable patients, these changes may compromise myocardial and cerebral perfusion [3]. Large perioperative cohort studies have shown that even short periods of intraoperative hypotension, particularly when mean blood pressure (MBP) falls below approximately 65 mmHg, are associated with postoperative myocardial injury, acute kidney injury (AKI), stroke, and increased mortality [4].

Tourniquet-release hypotension is likely multifactorial. Deflation abruptly decreases systemic vascular resistance, redistributes blood volume into the previously ischemic limb, and releases vasoactive metabolites such as adenosine, lactate, and potassium into the systemic circulation [2,5,6]. In elderly patients with impaired autonomic responses or diastolic dysfunction, these changes may lead to clinically important reductions in arterial pressure, especially when coronary and cerebral perfusion pressures are already marginal [3]. Despite its clinical relevance, anesthetic management around tourniquet release remains variable and is often guided more by individual practice than by validated risk stratification tools.

Dynamic preload indices such as the pleth variability index (PVi) have attracted attention as noninvasive markers of fluid responsiveness during controlled mechanical ventilation [7,8,9,10]. Higher PVi values have been associated with hypovolemia and increased likelihood of fluid responsiveness [11], and several studies have shown that elevated baseline PVi predicts hypotension after induction of general anesthesia, particularly in high-risk or geriatric patients [6,12,13,14]. However, the clinical utility of PVi in geriatric cohorts remains controversial, as age-related vascular stiffness, baseline vasoplegia, and the frequent administration of vasoactive medications can significantly alter peripheral perfusion waveforms and compromise the reliability of dynamic preload indices [15,16]. Currently, the utility of PVi for predicting hemodynamic instability during specific intraoperative events, such as tourniquet release during TKA, remains unclear. Although pneumatic thigh tourniquets are widely used in TKA, abrupt hemodynamic depression after deflation is highly prevalent and hazardous in geriatric patients due to physiological aging and cardiovascular comorbidities [2,3]. Traditional static hemodynamic monitors are often insufficient for predicting this sudden vasodilation-driven hypotension, and there is a critical clinical gap in simple, noninvasive, and dynamic predictors to stratify patient risk before tourniquet deflation. Our study addresses this clinical gap by evaluating a combined pre-release perfusion pressure and early dynamic volume index (intuPVi) bedside risk assessment framework under real-world clinical constraints.

Accordingly, we prospectively evaluated whether pre-release blood pressure and PVi could serve as useful predictors of tourniquet-release hypotension in elderly patients undergoing TKA under general anesthesia. We also present the findings with explicit acknowledgment of the limitations of intermittent noninvasive monitoring, exploratory secondary analyses, and modest sample size.

## 2. Materials and Methods

### 2.1. Study Design and Ethical Approval

This single-center prospective observational study was conducted at a tertiary care hospital. The institutional review board approved the study protocol, and written informed consent was obtained from all participants. The study was performed in accordance with the Declaration of Helsinki and applicable institutional and national regulations.

### 2.2. Study Population

Consecutive adult patients who underwent elective primary unilateral TKA under general anesthesia with a thigh tourniquet were screened. Inclusion criteria were age ≥ 65 years, elective unilateral TKA, use of a pneumatic thigh tourniquet with documented inflation and deflation times, and availability of noninvasive blood pressure and PVi measurements at predefined perioperative time points. Exclusion criteria were revision TKA or bilateral procedures, combined regional/general anesthesia with neuraxial block as the primary anesthetic, baseline arrhythmias that could invalidate dynamic preload indices, and missing hemodynamic or PVi data around the time of tourniquet release. Ninety patients met all criteria and were included in the final analysis.

### 2.3. Anesthesia and Tourniquet Management

Anesthesia was induced with intravenous propofol (1–2 mg/kg), followed by a neuromuscular blocking agent to facilitate endotracheal intubation. Anesthesia was maintained with volatile anesthetics in oxygen/air and continuous remifentanil infusion. Patients were mechanically ventilated in volume-controlled mode with a tidal volume of approximately 6–8 mL/kg. A pneumatic thigh tourniquet was applied to the operative limb and inflated after exsanguination. Tourniquet pressure was generally set at 100–150 mmHg above systolic blood pressure and maintained until completion of the major surgical steps. Tourniquet inflation and deflation times were recorded and converted to minutes to calculate tourniquet duration.

Intravenous fluids and vasoactive agents, including phenylephrine, ephedrine, or norepinephrine, were administered at the discretion of the attending anesthesiologist to maintain hemodynamic stability.

### 2.4. Monitoring

Noninvasive blood pressure (NIBP) was measured on the contralateral arm at 1–5 min intervals. For analysis, we abstracted systolic and mean blood pressure values at key time points: baseline (iniSBP and iniMBP) immediately before induction; pre-release systolic and mean blood pressure (preRel_SBP and preRel_MBP), defined as the last values recorded within 5 min before tourniquet deflation (befoffSBP and befoffMBP); and the minimum systolic and mean blood pressures after tourniquet deflation (offSBP and offMBP) within a precisely defined post-deflation window of 10 min following tourniquet deflation.

PVi was measured using a Radical-7 monitor with a rainbow sensor (Masimo Corp., Irvine, CA, USA). PVi values were recorded immediately before induction (iniPVi), immediately after endotracheal intubation (intuPVi), at or near tourniquet inflation (onPVi), during surgery at predefined intervals (inciPVi, 1hPVi), immediately before deflation (befoffPVi), and shortly after deflation (offPVi). Preliminary analyses from the pilot study suggested that PVi measured immediately after intubation (intuPVi) showed the clearest relationship with post-deflation blood pressure; therefore, intuPVi was selected as the primary PVi predictor in the present analysis.

### 2.5. Data Collection and Outcomes

Demographic and clinical variables were extracted from electronic medical records, including age, sex, body mass index (BMI), American Society of Anesthesiologists (ASA) physical status, history of hypertension (HTN) and diabetes mellitus (DM), anesthesia duration, surgery duration, tourniquet duration, crystalloid volume, and estimated blood loss (EBL).

The primary outcome was tourniquet-release hypotension defined as offMBP < 65 mmHg (Severe_offMBP65), in line with prior large cohort studies linking intraoperative MBP < 65 mmHg to adverse outcomes [4]. Two secondary hypotension outcomes were evaluated for comparison: an absolute systolic definition (Severe_offSBP90, offSBP < 90 mmHg) and a relative systolic definition (Severe_pre20), defined as a ≥20% decrease in systolic blood pressure from pre-release values to the nadir after deflation. These secondary endpoints were included to examine whether the observed associations were consistent across commonly used absolute and relative blood pressure definitions and should be interpreted as supportive exploratory analyses rather than primary inferential outcomes.

Postoperative outcomes of interest included postoperative nausea and vomiting (PONV), wound infection, postoperative pulmonary complication (PPC), urinary retention (need for Nelaton catheterization and postoperative urinary retention [POUR]), AKI, delirium, postoperative creatinine, glomerular filtration rate (GFR), and hospital length of stay (HLOS).

### 2.6. Sample Size Calculation and Statistical Analysis

Based on a pilot study conducted at our institution in patients undergoing TKA, the correlation coefficient between the degree of blood pressure decrease at tourniquet release and PVi was 0.493. A G*Power (version 3.1; Heinrich-Heine-Universität Düsseldorf, Düsseldorf, Germany) analysis (Exact test, Correlation: bivariate normal model, a priori computation of required sample size, two-tailed, correlation ρH1 = 0.493, α error probability = 0.01, power = 0.99, correlation ρH0 = 0) yielded a required sample size of 85 patients. Assuming a 5% dropout rate, we set the target enrollment at 90 patients. We acknowledge that while this sample size was calculated a priori based on the correlation coefficient as a primary surrogate of association, it was not specifically powered for higher-dimensional multivariable logistic regression or receiver operating characteristic (ROC) analysis, representing a limitation in statistical depth.

Continuous variables are reported as mean ± standard deviation (SD) or median (interquartile range), as appropriate. Normality was assessed using the Shapiro–Wilk test and visual inspection of histograms. Between-group comparisons for the primary endpoint were performed using the Mann–Whitney U test for continuous variables and the chi-square or Fisher’s exact test for categorical variables. Univariate logistic regression analyses were first used to examine associations between each hypotension endpoint and clinically plausible predictors. Parsimonious multivariable logistic regression models were then constructed for each hypotension endpoint, including pre-release blood pressure, intuPVi, and HTN. Model performance was assessed using receiver operating characteristic analysis and the area under the curve (AUC). All statistical tests were two-sided, and *p* < 0.05 was considered statistically significant. Statistical analyses were performed using SPSS version 22.0 (IBM Corp., Armonk, NY, USA).

## 3. Results

### 3.1. Patient Characteristics and Incidence of Tourniquet-Release Hypotension

A total of 90 elderly patients met the inclusion criteria and were included in the final analysis. Most patients were female (86.7%), and ASA physical status II was the most common classification (80.0%). The mean age was 73.88 ± 5.37 years, and the mean body mass index was 25.74 ± 3.03 kg/m^2^. HTN and DM were present in 64.4% and 34.4% of patients, respectively. Baseline SBP and MBP were 171.18 ± 21.37 mmHg and 118.04 ± 14.90 mmHg, respectively. Pre-release SBP and MBP were 129.67 ± 19.89 mmHg and 94.33 ± 14.39 mmHg, whereas the nadir post-deflation SBP and MBP were 96.30 ± 14.11 mmHg and 70.87 ± 10.00 mmHg, respectively (Table A1).

Tourniquet-release hypotension, defined as offMBP < 65 mmHg, occurred in 26 of 90 patients (28.9%). The incidence of offSBP < 90 mmHg was 35.6%, and the incidence of a ≥20% decrease in SBP from pre-release values was 72.2%.

Baseline and perioperative characteristics according to the presence of offMBP < 65 mmHg are summarized in Table 1. Patients with hypotension had significantly lower pre-release SBP and MBP than those without hypotension. Body mass index was also slightly lower in the hypotension group, whereas age, anesthesia duration, surgery duration, tourniquet duration, crystalloid volume, and estimated blood loss did not differ significantly between groups (Table 1).

### 3.2. Predictors of the Primary Endpoint

Univariate logistic regression analysis showed that lower pre-release MBP was significantly associated with offMBP < 65 mmHg (odds ratio (OR), 0.94; 95% confidence interval (CI), 0.91–0.98; *p* = 0.003). Lower pre-release SBP was also associated with the primary endpoint (OR, 0.96; 95% CI, 0.94–0.99; *p* = 0.007). PVi measured immediately after endotracheal intubation (intuPVi) showed a borderline association with the primary endpoint (OR, 1.12; 95% CI, 1.00–1.25; *p* = 0.042). Other candidate predictors were not significantly associated with the primary endpoint (Table 2).

**Figure 1:**
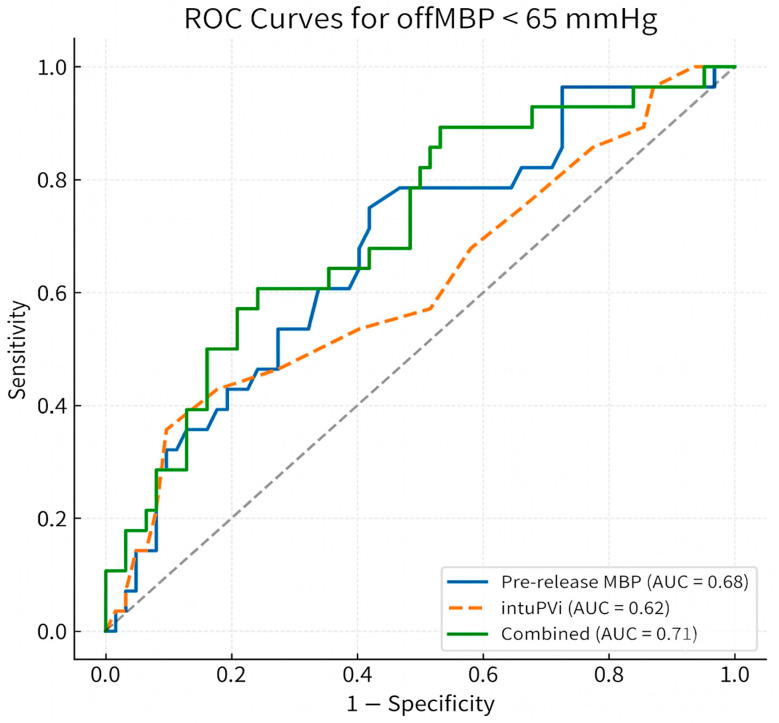
Receiver operating characteristic (ROC) curves for predicting tourniquet-release hypotension, defined as offMBP < 65 mmHg. Curves are shown for pre-release mean blood pressure (MBP) alone, PVi measured immediately after endotracheal intubation (intuPVi) alone, and a combined logistic regression model including both predictors. The grey dotted line represents the reference line (AUC = 0.5).

In the multivariable model, pre-release MBP and intuPVi remained independently associated with offMBP < 65 mmHg. The adjusted OR was 0.95 (95% CI, 0.92–0.99; *p* = 0.012) for pre-release MBP and 1.12 (95% CI, 1.00–1.26; *p* = 0.042) for intuPVi. We acknowledge that the association between lower pre-release MBP and post-deflation hypotension is partly expected and mathematically related. However, including pre-release MBP in our multivariable model was essential to demonstrate that the dynamic index (intuPVi) provides independent and additive predictive information beyond the absolute blood pressure level immediately before deflation. The discriminatory performance of the combined model was moderate, with an AUC of 0.71 (Table 3 and Figure 1).

### 3.3. Secondary Blood Pressure Outcomes

For the secondary hypotension definitions, similar patterns were observed. Pre-release SBP and intuPVi were significantly associated with offSBP < 90 mmHg in the parsimonious multivariable model. In addition, both variables were associated with a ≥20% decrease in SBP from pre-release values to the post-deflation nadir (Table 3).

The combined model showed an AUC of 0.76 for offSBP < 90 mmHg and 0.88 for the relative SBP decrease endpoint, indicating that pre-release SBP was particularly informative for the relative definition, whereas intuPVi provided modest additional discrimination (Table 3).

### 3.4. Exploratory Bedside Risk Stratification

**Figure A1:**
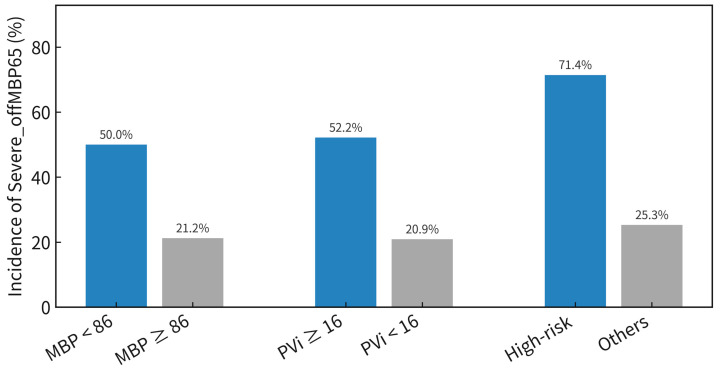
Simplified bar chart showing the incidence of offMBP < 65 mmHg according to pre-release MBP (≥86 vs. <86 mmHg), intuPVi (<16 vs. ≥16), and a combined high-risk group (pre-release MBP < 86 mmHg and intuPVi ≥ 16). Risk strata (MBP < 86 mmHg, PVi ≥ 16, and the combined high-risk group) are shown as blue bars on the left of each pair.

An exploratory bedside rule was assessed using thresholds of pre-release MBP < 86 mmHg and intuPVi ≥ 16. Patients with pre-release MBP < 86 mmHg had a higher incidence of offMBP < 65 mmHg than those with pre-release MBP ≥ 86 mmHg (50.0% vs. 21.2%). Similarly, patients with intuPVi ≥ 16 had a higher incidence of offMBP < 65 mmHg than those with intuPVi < 16 (52.2% vs. 20.9%). When both criteria were present, the incidence of offMBP < 65 mmHg was 71.4%, compared with 25.3% in all other patients. Because only seven patients met both criteria, this analysis should be interpreted as exploratory. The corresponding visual summary is provided in the Appendix B as Figure A1.

### 3.5. Postoperative Outcomes

Postoperative outcomes according to the presence of offMBP < 65 mmHg are presented in Table 4. The incidences of PONV, wound infection, PPC, urinary retention, AKI, and delirium were similar between groups. Postoperative day 2 creatinine and GFR also did not differ significantly. HLOS was numerically shorter in patients with offMBP < 65 mmHg, but the difference was not statistically significant (Table 4).

Overall, no clear association was observed between tourniquet-release hypotension and early postoperative complications in this cohort.

## 4. Discussion

This prospective study in elderly patients undergoing TKA with a thigh tourniquet under general anesthesia demonstrates that pre-release MBP and PVi immediately after endotracheal intubation (intuPVi) are independent predictors of tourniquet-release hypotension when defined as offMBP < 65 mmHg. Similar associations were observed when hypotension was defined using a systolic threshold (offSBP < 90 mmHg) and a relative decrease of ≥20% from pre-release to nadir SBP after deflation. These findings suggest that both the blood pressure level immediately before tourniquet deflation and the degree of preload dependence early after induction jointly determine vulnerability to tourniquet-release hypotension in this population.

The association between pre-release MBP and Severe_offMBP65 is clinically intuitive and consistent with prior large studies showing that intraoperative MBP below approximately 65 mmHg is associated with myocardial and renal injury [4,17,18,19]. In our cohort, lower pre-release MBP was associated with increased odds of offMBP < 65 mmHg, even after adjustment for HTN and intuPVi. This reinforces the concept that allowing the MBP to drift too low immediately before tourniquet release may be hazardous in elderly patients. At the same time, we acknowledge that this predictor is mathematically and mechanistically expected (since patients starting with lower blood pressure naturally require less of a decrease to fall below the absolute offMBP threshold) and therefore offers pragmatic bedside relevance more than conceptual novelty. However, its inclusion in the multivariable model establishes that intuPVi exerts an independent predictive effect that is not merely a surrogate for low baseline pressure.

IntuPVi also emerged as an independent predictor of Severe_offMBP65 in the multivariable model. Elevated PVi has been associated with hypovolemia, increased vascular capacitance, and limited hemodynamic reserve, and higher baseline or pre-induction PVi predicts hypotension after anesthesia induction in several studies [12,13,14]. Tsuchiya et al. showed that pre-anesthesia PVi strongly correlated with the MAP decrease during propofol induction and that PVi ≥ 15% identified patients at risk of a marked MAP drop [13]. These data support the concept that higher PVi marks a circulation that is more preload dependent and less able to tolerate vasodilation.

In our study, PVi was measured shortly after endotracheal intubation, during a period when induction-related vasodilation and venous pooling coexist with strong sympathetic stimulation from laryngoscopy. Under these competing influences, a persistently elevated PVi is unlikely to be explained solely by transient sympathetic activation. Rather, it likely reflects a circulation with reduced autonomic and vascular reserve in which induction-induced sympathetic inhibition and increased vascular capacitance have not been fully compensated. Viewed in this way, intuPVi functions as a surrogate of baseline autonomic/vascular reserve. Our finding that intuPVi independently predicted tourniquet-release hypotension extends previous work on induction hypotension [12,13,14] by showing that an early PVi measurement can also identify patients who are vulnerable to a later, discrete intraoperative event like tourniquet release. Nevertheless, because befoffPVi was not associated with the primary endpoint, this interpretation remains inferential and should not be overstated; an alternative explanation is that the intuPVi finding reflects exploratory multiple testing with this sample size.

The combined use of pre-release blood pressure and intuPVi improved the prediction of tourniquet-release hypotension compared with either variable alone, although the absolute gains in AUC were modest. Nevertheless, the overall discrimination of the parsimonious models was moderate for a simple bedside risk assessment tool. Using pre-release MBP < 86 mmHg and intuPVi ≥ 16 as pragmatic thresholds, we identified a high-risk subgroup in which Severe_offMBP65 occurred in 71.4%, compared with 25.3% in all other patients. This gradient in risk suggests that a two-dimensional assessment incorporating both the absolute perfusion pressure immediately before deflation (pre-release MBP) and a dynamic index of preload dependency and vascular reserve (intuPVi) may be more informative than either parameter alone for stratifying risk of tourniquet-release hypotension. Because model discrimination was only moderate and the high-risk subgroup was small, we propose this approach as a hypothesis-generating framework for future validation rather than as an immediate trigger for protocolized intervention.

Despite these clear intraoperative associations, we did not observe strong or consistent associations between tourniquet-release hypotension (as defined by offMBP < 65 mmHg or the alternative SBP-based definitions), intuPVi, and early postoperative complications such as PONV, wound infection, PPC, urinary retention, AKI, delirium, or short-term changes in creatinine and GFR. The absence of clear signals in these outcomes likely reflects the modest sample size and low event rates [4,17,18,19] rather than the absence of any causal link between intraoperative hypotension and postoperative morbidity. Large multicenter studies using time-weighted measures of hypotension and rigorous adjudication of myocardial and renal injury have already established such links. Our study should therefore be viewed as preliminary, focusing on the identification of simple, noninvasive intraoperative predictors of tourniquet-release hypotension rather than definitive outcome relationships.

We did not compare different anesthetic techniques, as all patients in our cohort received a standardized general anesthetic protocol consisting of propofol induction and volatile maintenance. In the literature, the choice between total intravenous anesthesia (TIVA) with propofol and volatile anesthesia has shown conflicting results regarding the severity of tourniquet-release hypotension, with some studies suggesting propofol preserves vascular tone slightly better, while others report no clinical difference [20,21]. Furthermore, regional anesthesia (e.g., spinal or epidural anesthesia) is also commonly used in TKA and is generally associated with lower baseline vascular resistance due to sympathetic blockade, which may alter the hemodynamic response and the predictive accuracy of dynamic indices like PVi [22].

We also evaluated the potential of sex as a predictor of tourniquet-release hypotension. In our cohort, sex distribution was identical between the hypotension and non-hypotension groups (*p* = 1.000, Table 1), indicating that sex was not a significant predictor in this cohort. The high prevalence of female patients (86.7%) in our study reflects the established epidemiological characteristics of knee osteoarthritis in South Korea, which is highly prevalent in older women [23,24]. Although sex-specific physiological differences in vascular compliance and autonomic responses could theoretically influence perioperative hemodynamic shifts, our study was not powered to detect subtle sex-based differences due to the small proportion of male participants.

This study has several limitations. Its prospective, single-center design limits generalizability and introduces the possibility of residual confounding by unmeasured variables such as anesthetic depth, vasoactive drug dosing, and postoperative care pathways. The sample size of 90 patients, while adequate to detect moderate associations between pre-release blood pressure, PVi, and tourniquet-release hypotension based on our a priori power calculation, is relatively small for multivariable modeling of less frequent postoperative complications. With 26 primary hypotension events, our multivariable model is susceptible to overfitting, which may inflate the predictive performance. We evaluated multiple PVi time points without formal statistical adjustment (e.g., Bonferroni correction) for multiple comparisons, and selected intuPVi as the primary predictor based on preliminary analyses; this exploratory approach carries an inherent risk of post hoc variable selection bias. Furthermore, intraoperative fluid management and vasopressor administration were left to the discretion of the attending anesthesiologists. This lack of a standardized hemodynamic protocol introduces confounding, as proactive vasoactive administration could mask or attenuate tourniquet-release hypotension, while baseline vasoplegia in the elderly may limit PVi utility [8,16]. Finally, our hypotension definitions were based on single nadir values and did not capture the duration or cumulative burden of hypotension, which may be more relevant to organ injury [17,18,19]. In addition, we acknowledge that standard intermittent NIBP monitoring (every 1–5 min) rather than continuous invasive arterial pressure monitoring represents a clinical limitation because it lacks the temporal resolution to capture very brief or rapid hemodynamic nadirs immediately following tourniquet release. However, because standard TKA is routinely performed under noninvasive monitoring in real-world clinical practice, this constraint is precisely what motivated our study. Since NIBP cannot continuously monitor post-deflation trends, establishing pre-release predictors (such as early intuPVi and pre-release MBP) provides a pragmatic bedside risk-stratification framework to proactively identify patients at risk before tourniquet deflation occurs.

Despite these limitations, our findings suggest that pre-release MBP and PVi could serve as preliminary indicators to help identify elderly TKA patients who may be vulnerable to tourniquet-release hypotension. In a research context, this underscores the potential value of examining pre-release hemodynamic states rather than relying solely on post-deflation reactive measures. However, translating these findings into clinical practice—such as implementing proactive hemodynamic protocols or preemptively adjusting vasopressor dosing before tourniquet deflation—would be premature without prospective validation in larger, standardized cohorts. Future prospective multicenter studies should validate these observations, examine whether targeted interventions based on pre-release blood pressure and PVi can reduce the incidence and duration of tourniquet-release hypotension, and determine whether such strategies can ultimately improve patient-centered outcomes.

## 5. Conclusions

Lower pre-release MBP and higher intuPVi were independently associated with tourniquet-release hypotension in elderly patients undergoing TKA under general anesthesia. These findings are hypothesis-generating and suggest that these variables may be useful for preliminary peri-deflation risk stratification. However, the findings must be interpreted cautiously because of the moderate model performance, intermittent noninvasive monitoring, post hoc selection, and the modest sample size. External validation in larger cohorts using standardized hemodynamic management protocols is required before these markers can be integrated into routine clinical practice.

## Figures and Tables

**Table 1:** Baseline characteristics and perioperative data according to tourniquet-release hypotension, defined as offMBP < 65 mmHg.

Variable	Non-Hypotension (*n* = 64)	Hypotension (*n* = 26)	*p*-Value
Sex (male/female)	9/55	3/23	1.000
ASA class 1/2/3	13/47/4	1/25/0	0.049
Hypertension	38 (59.4%)	20 (76.9%)	0.115
Diabetes mellitus	23 (35.9%)	8 (30.8%)	0.640
Age (years)	73.31 ± 5.59	75.27 ± 4.58	0.072
BMI (kg/m^2^)	26.04 ± 2.87	25.01 ± 3.34	0.045
Baseline SBP (mmHg)	169.80 ± 21.42	174.58 ± 21.26	0.308
Baseline MBP (mmHg)	117.78 ± 13.85	118.69 ± 17.49	0.782
Pre-release SBP (mmHg)	133.39 ± 20.22	120.50 ± 15.96	0.003
Pre-release MBP (mmHg)	97.38 ± 14.16	86.85 ± 12.23	0.001
offSBP (mmHg)	102.16 ± 11.82	81.88 ± 7.26	<0.001
offMBP (mmHg)	75.48 ± 7.62	59.50 ± 4.56	<0.001
Anesthesia time (min)	143.05 ± 13.44	142.31 ± 17.68	0.391
Operation time (min)	101.47 ± 12.03	99.85 ± 14.87	0.532
Tourniquet time (min)	93.70 ± 8.59	90.88 ± 10.63	0.145
Crystalloid volume (mL)	321.88 ± 110.15	280.77 ± 105.90	0.117
Estimated blood loss (mL)	42.66 ± 21.98	38.46 ± 24.12	0.223

Values are presented as the mean ± SD or number (%). ASA, American Society of Anesthesiologists; BMI, body mass index; SBP, systolic blood pressure; MBP, mean blood pressure.

**Table 2:** Univariate predictors of tourniquet-release hypotension, defined as offMBP < 65 mmHg.

Predictor	OR (95% CI)	*p*-Value
Pre-release MBP (mmHg)	0.94 (0.91–0.98)	0.003
Pre-release SBP (mmHg)	0.96 (0.94–0.99)	0.007
intuPVi	1.12 (1.00–1.25)	0.042
inciPVi	1.10 (0.99–1.22)	0.085
Crystalloid volume (mL)	1.00 (0.99–1.00)	0.111
Hypertension	2.28 (0.81–6.45)	0.120
Age (years)	1.07 (0.98–1.17)	0.120
BMI (kg/m^2^)	0.89 (0.75–1.04)	0.147
Tourniquet time (min)	0.97 (0.92–1.02)	0.192
onPVi	1.06 (0.95–1.19)	0.290
Baseline SBP (mmHg)	1.01 (0.99–1.03)	0.336
Cerebrovascular disease	1.96 (0.41–9.43)	0.403
Cardiac disease	1.76 (0.45–6.83)	0.415
Estimated blood loss (mL)	0.99 (0.97–1.01)	0.428
Surgical time (min)	0.99 (0.95–1.03)	0.586
Diabetes mellitus	0.79 (0.30–2.10)	0.640
1hPVi	1.01 (0.94–1.09)	0.707
offPVi	1.02 (0.91–1.14)	0.771
Baseline MBP (mmHg)	1.00 (0.97–1.04)	0.792
iniPVi	1.01 (0.95–1.06)	0.815
befoffPVi	1.01 (0.93–1.10)	0.820
Anesthesia time (min)	1.00 (0.97–1.03)	0.828

OR, odds ratio; CI, confidence interval; BMI, body mass index; MBP, mean blood pressure; SBP, systolic blood pressure; PVi, pleth variability index.

**Table 3:** Parsimonious multivariable models for different definitions of tourniquet-release hypotension.

Outcome	Predictor	Adjusted OR (95% CI)	*p*-Value	Model AUC
offMBP < 65 mmHg	Pre-release MBP	0.95 (0.92–0.99)	0.012	0.71
	intuPVi	1.12 (1.00–1.26)	0.042	
offSBP < 90 mmHg	Pre-release SBP	0.96 (0.94–0.99)	0.006	0.76
	intuPVi	1.15 (1.02–1.29)	0.018	
≥20% decrease in SBP	Pre-release SBP	1.12 (1.06–1.18)	<0.001	0.88
	intuPVi	1.20 (1.02–1.40)	0.029	

OR, odds ratio; CI, confidence interval; MBP, mean blood pressure; SBP, systolic blood pressure; PVi, pleth variability index; AUC, area under the receiver operating characteristic curve.

**Table 4:** Postoperative outcomes according to tourniquet-release hypotension, defined as offMBP < 65 mmHg.

Outcome	Non-Hypotension (*n* = 64)	Hypotension (*n* = 26)	*p*-Value
PONV	27/63 (42.9%)	9/25 (36.0%)	0.555
Wound infection	1/64 (1.6%)	0/26 (0.0%)	1.000
PPC	4/64 (6.2%)	1/26 (3.8%)	1.000
Nelaton catheterization	35/64 (54.7%)	15/26 (57.7%)	0.795
POUR	40/64 (62.5%)	16/26 (61.5%)	0.932
AKI	0/64 (0.0%)	1/26 (3.8%)	0.289
Delirium	8/64 (12.5%)	5/26 (19.2%)	0.510
CrPOD2	0.62 ± 0.26	0.61 ± 0.18	0.627
GFRPOD2	110.00 ± 33.56	108.31 ± 34.76	0.454
HLOS	9.95 ± 4.10	8.58 ± 3.92	0.167

PONV, postoperative nausea and vomiting; PPC, postoperative pulmonary complication; POUR, postoperative urinary retention; AKI, acute kidney injury; CrPOD2, creatinine on postoperative day 2; GFRPOD2, glomerular filtration rate on postoperative day 2; HLOS, hospital length of stay.

## Data Availability

Data can be obtained from the authors upon reasonable request.

## Abbreviations

The following abbreviations are used in this manuscript:

AKI	Acute kidney injury
ASA	American Society of Anesthesiologists
AUC	Area under the curve
BMI	Body mass index
CI	Confidence interval
DM	Diabetes mellitus
EBL	Estimated blood loss
GFR	Glomerular filtration rate
HLOS	Hospital length of stay
HTN	Hypertension
MBP	Mean blood pressure
OR	Odds ratio
PONV	Postoperative nausea and vomiting
POUR	Postoperative urinary retention
PPC	Postoperative pulmonary complication
PVi	Pleth variability index
ROC	Receiver operating characteristic
SBP	Systolic blood pressure
TKA	Total knee arthroplasty

## Appendix A

**Table A1:** Baseline characteristics of study participants.

Variable	Value (*n* = 90)
Female	78 (86.7%)
ASA class II	72 (80.0%)
Age (years)	73.88 ± 5.37
BMI (kg/m^2^)	25.74 ± 3.03
Hypertension	58 (64.4%)
Diabetes mellitus	31 (34.4%)
Baseline SBP (mmHg)	171.18 ± 21.37
Baseline MBP (mmHg)	118.04 ± 14.90
Pre-release SBP (mmHg)	129.67 ± 19.89
Pre-release MBP (mmHg)	94.33 ± 14.39
offSBP (mmHg)	96.30 ± 14.11
offMBP (mmHg)	70.87 ± 10.00
Anesthesia time (min)	142.83 ± 14.69
Operation time (min)	101.00 ± 12.85
Tourniquet time (min)	92.89 ± 9.25
